# ﻿*Thismiaaliasii* (Thismiaceae), a new species from Terengganu, Peninsular Malaysia

**DOI:** 10.3897/phytokeys.254.136085

**Published:** 2025-03-31

**Authors:** Mat Yunoh Siti-Munirah, Shakri Mohamad Alias

**Affiliations:** 1 Forest Research Institute Malaysia, 52109 Kepong, Selangor, Malaysia Forest Research Institute Malaysia Kepong Malaysia; 2 Jabatan Perhutanan Negeri Terengganu, 21500 Kuala Terengganu, Terengganu, Malaysia Jabatan Perhutanan Negeri Terengganu Kuala Terengganu Malaysia

**Keywords:** Achlorophyllous plants, endemism, Gunung Chemerong, monocots, Terengganu, *Thismia* subsection *Odoardoa*

## Abstract

A new mycoheterotrophic species, *Thismiaaliasii*, is described and illustrated. This species inhabits a hill dipterocarp forest in mountains of eastern Peninsular Malaysia. *Thismiaaliasii* differs from other *Thismia* species by the following features: tepals equal in size and shape with different length of their appendages, appendages of the outer tepals shorter than those of the inner tepals (3.5 mm vs. ca. 26–32 mm long), stamen supraconnective at apex with three long filiform appendages and two acute appendages, and the margins of individual connectives abaxially raised into a conspicuous rib. With respect to floral morphology, *T.aliasii* should be placed to Thismiasubsect.Odoardoa. According to the categories and criteria of the IUCN Red List, *T.aliasii* is provisionally classified as Critically Endangered (CR).

## ﻿Introduction

The fully mycoheterotrophic monocot family Thismiaceae is mainly confined to tropical and subtropical regions. Species of the family are small herbs that usually inhabit shady, humid environments, often hidden under the leaf litter (Merckx et al. 2024). The genus *Thismia* Griff., often referred to as "fairy lanterns" (Wapstra et al. 2005), is remarkable due to its unusual flower appearance. The unique flower design of the genus is related to the specialized pollination mechanisms that involve small insects such as fungus gnats (belonging to the genus *Corynoptera*) (Guo et al. 2019). *Thismia* flowers were also visited and possibly pollinated by a wide range of other invertebrates, such as scuttle flies (Phoridae, Diptera) (Yudina et al. 2021).

The genus *Thismia* comprises about 116 species (Besi et al. 2024; Chung et al. 2024; Chunyang et al. 2024; Nuraliev and Sennikov 2024; POWO 2024; Siti-Munirah et al. 2024; Ya et al. 2024). It is the most widespread and species-rich genus within the family and has a high degree of endemism. Its range extends from tropical and subtropical Asia to northern and eastern Australia and New Zealand, and from Costa Rica to tropical South America with an isolated occurrence in North America (POWO 2024). Many of its species are considered extremely rare, with scattered distributions, and some may already be extinct, but there are also species with quite wide ranges and multiple known localities (Dančák et al. 2020).

**Figure 1.**
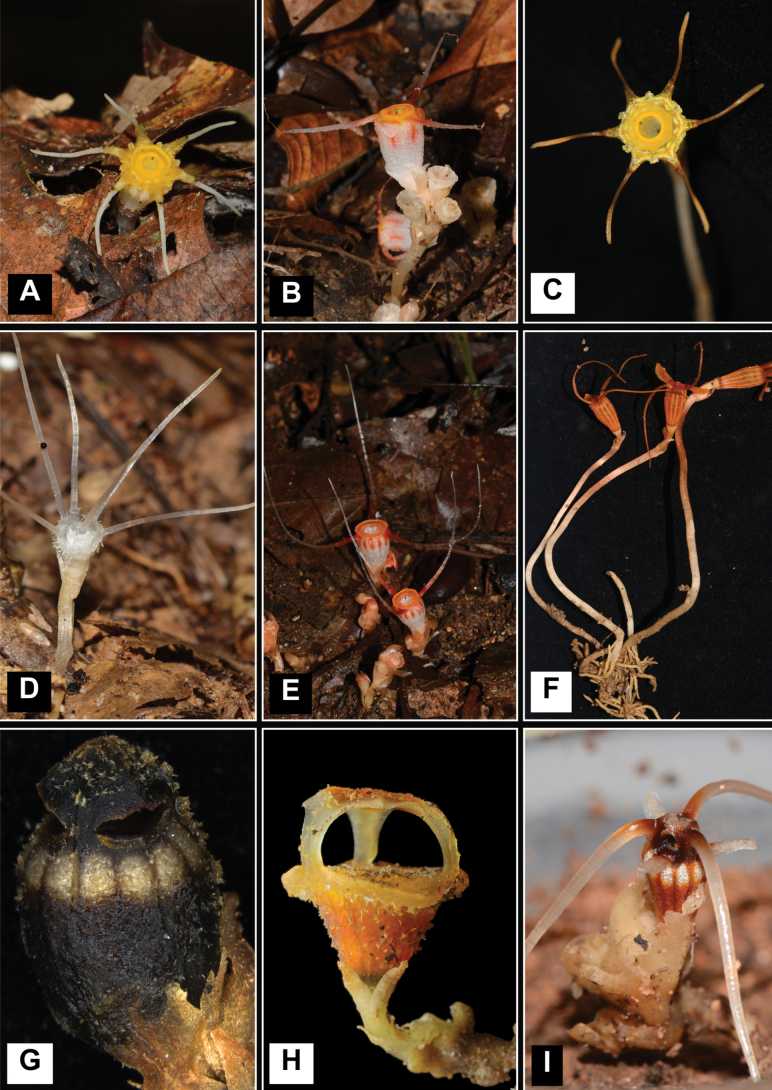
*Thismia* species occurring in the state of Terengganu **A***Thismiaalba***B***T.arachnites***C***T.aseroe***D***T.domei***E***T.javanica***F***T.kenyirensis***G***T.latiffiana***H***T.sitimeriamiae***I***T.terengganuensis*. Photos by Siti-Munirah (**A–G**, **I**) and Dome (**H**).

**Figure 2.**
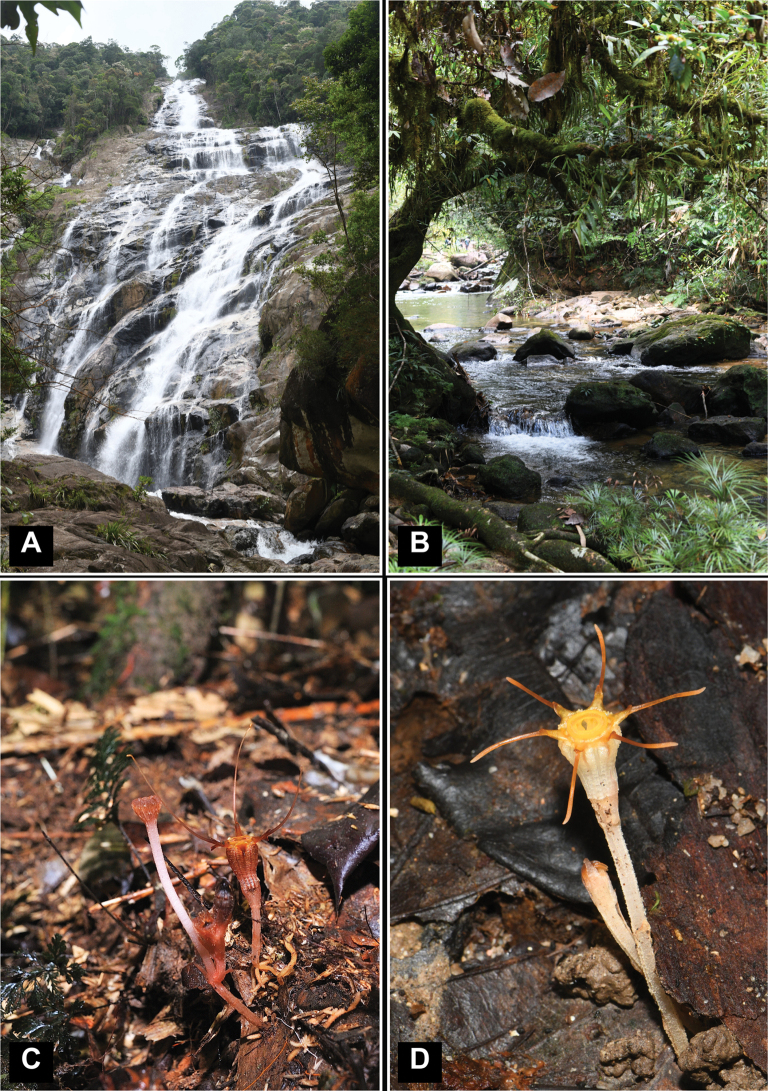
Landscapes of Gn. Chemerong and the species of *Thismia* found there **A** the Chemerong waterfall with a height of 370 m **B** the Chemerong River not far from the habitat of *T.aliasii***C***Thismiaaliasii*, an individual discovered by Mohamad Alias in 2019 (FRI 91119) **D***Thismiaaseroe* (FRI 79116) found on the trail at Hutan Lipur Chemerong. Photos by Siti-Munirah (**A, B, D**) and Mohamad Alias (**C**).

The state of Terengganu is currently known to be the richest of the Peninsular Malaysian states in the species diversity of *Thismia* (Table 1, Fig. 1). All the 13 species known from Terengganu are present in the northern part of the state. The southern Terengganu, in contrast, is known to be inhabited by only two species of *Thismia*, both found in the vicinity of hill and mountain range known as Gunung (Gn.) Chemerong, which is part of the Hutan Lipur Gn. Chemerong (HLGC) or Chemerong Forest Eco Park and also well known as Chemerong-Berembun-Langsir (CBL) mountain range. This area is, in turn, a part of the Pasir Raja Forest Reserve (FR). One of these two species is *T.aseroe* (Fig. 2D), whereas the other one is *T.aliasii* described here as new to science.

**Table 1.** *Thismia* of Terengganu state (Siti-Munirah and Dome 2019, 2021, 2022, 2023a, 2023b; Siti-Munirah et al. 2021).

No.	Species	Locality in Terengganu	Status of Endemism
1.	*T.alba* Holttum ex Jonker	Taman Negeri Kenyir, Tembat FR	No
2.	*T.aliasii* Siti-Munirah	Gn. Chemerong (Pasir Raja FR)	Endemic
3.	*T.arachnites* Ridl.	Taman Negeri Kenyir	No
4.	*T.aseroe* Becc.	Gn. Chemerong (Pasir Raja FR)	No
5.	*T.brunneomitroides* Suetsugu & Tsukaya	Taman Negeri Kenyir	No
6.	*T.clavigeroides* Chantanaorr. & Seelanan	Taman Negeri Kenyir	No
7.	*T.domei* Siti-Munirah	Hulu Telemong FR	Endemic
8.	T. *javanica* J.J.Sm.	Hulu Telemong FR, Tembat FR	No
9.	*T.kenyirensis* Siti-Munirah & Dome	Taman Negeri Kenyir	Endemic
10.	*T.latiffiana* Siti-Munirah & Dome	Gn. Sarut (Hulu Nerus FR)	Endemic
11.	*T.ornata* Dančák, Hroneš & Sochor	Gn. Padang	No
12.	*T.sitimeriamiae* Siti-Munirah, Dome & Thorogood	Gn. Sarut (Hulu Nerus FR)	Endemic
13.	*T.terengganuensis* Siti-Munirah	Hulu Telemong FR	Endemic

## ﻿Materials and methods

**Figure 3.**
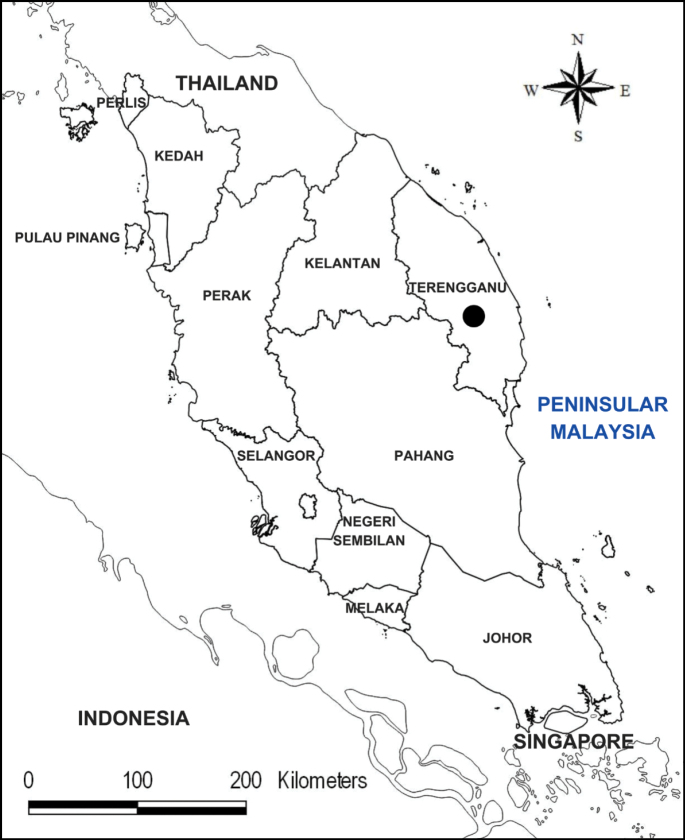
Gunung Chemerong (black circle) in Terengganu, the only known locality of *Thismiaaliasii*.

The assessment is based on the material collected in Gn. Chemerong, Hulu Terengganu, Terengganu (Fig. 3). *Thismiaaliasii* was first discovered during a field trip of the second author to CBL in 2019. The specimens were preserved in 70% ethanol and stored in the Kepong Herbarium (KEP). Three flowering individuals and one fruiting plant were collected and studied in total. Morphological characteristics were examined and measurements performed using an Olympus SZ61 stereomicroscope and high-resolution macrophotography. The measurements were made on fresh and spirit material.

## ﻿Taxonomic account

### ﻿ Thismia aliasii

Taxon classificationPlantaeDioscorealesBurmanniaceae

Siti-Munirah sp. nov.

5A527BBD-3783-531E-8BDE-11ED7CC76107

urn:lsid:ipni.org:names:77359488-1

Figs 4 , 5 , 6

#### Diagnosis.

*Thismiaaliasii* is very similar to the species of the T.subsectionOdoardoa, as the tepals are the same in shape and size. However, the tepal appendages of the new species are of unequal length, the inner ones are longer than the outer ones, while the tepal appendages of the other species are of equal length. In addition, in the new species the margins of the individual connectives are raised abaxially into the conspicuous rib, whereas connectives are almost flat abaxially in the rest of the species.

**Figure 4.**
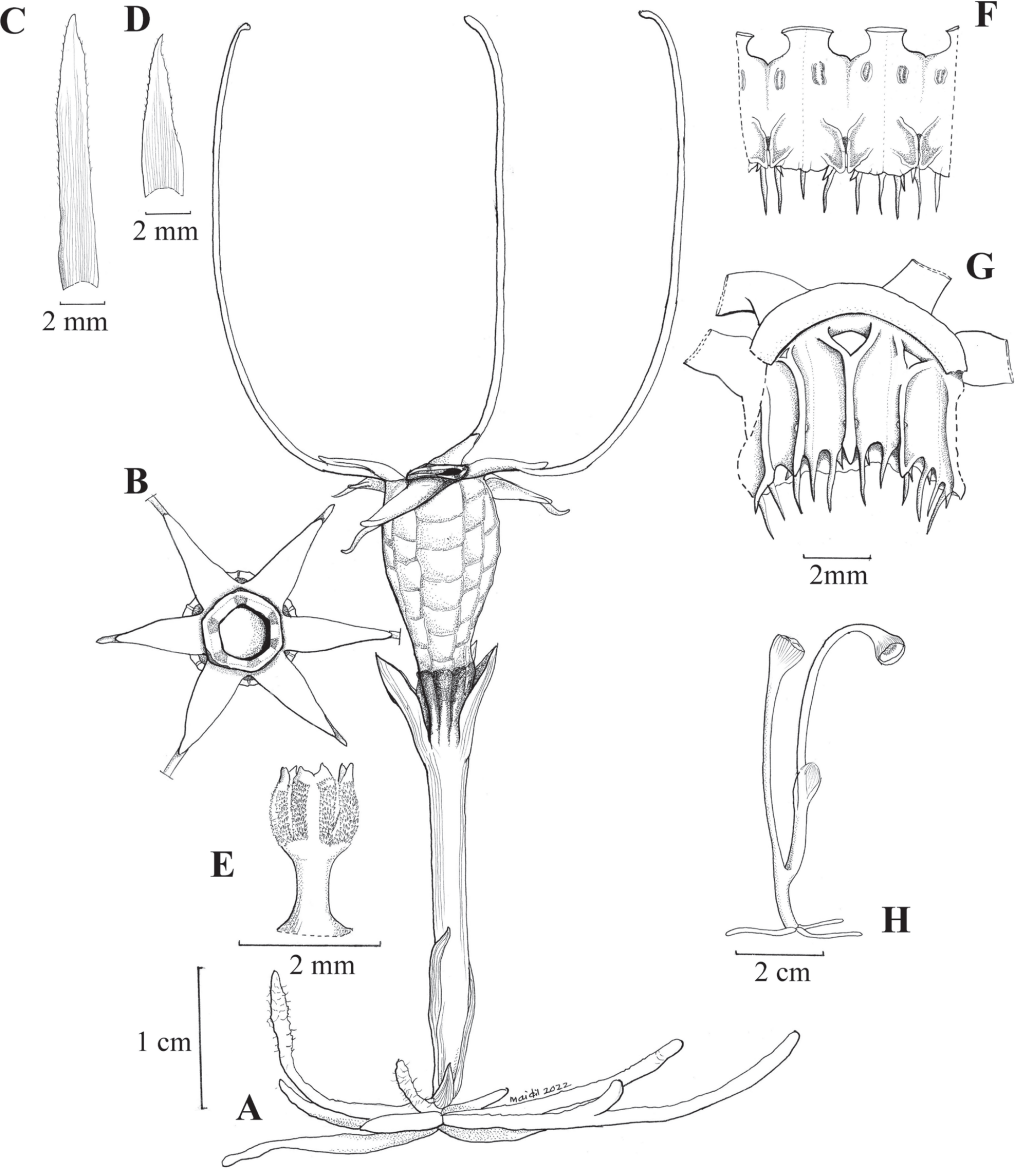
*Thismiaaliasii***A** plant with flower and roots **B** top view of flower showing tepals and annulus **C** bract (abaxial view) **D** leaf (abaxial view) **E** style and stigma **F** stamens (outer view) **G** stamens (inner view) **H** fruiting plant. All drawn by Mohamad Aidil Noordin from spirit material, FRI 91119.

#### Type.

Malaysia. • Peninsular Malaysia: Terengganu, Hulu Terengganu District, Hutan Simpan Pasir Raja, Chemerong Forest Eco Park, Gunung Chemerong, 4°39'33.2"N, 102°58'58.6"E, elev. ca 640 m, 26 July 2023, *Siti-Munirah, FRI 79119* (holotype KEP!, spirit collection, barcode no. SC13201).

**Figure 5.**
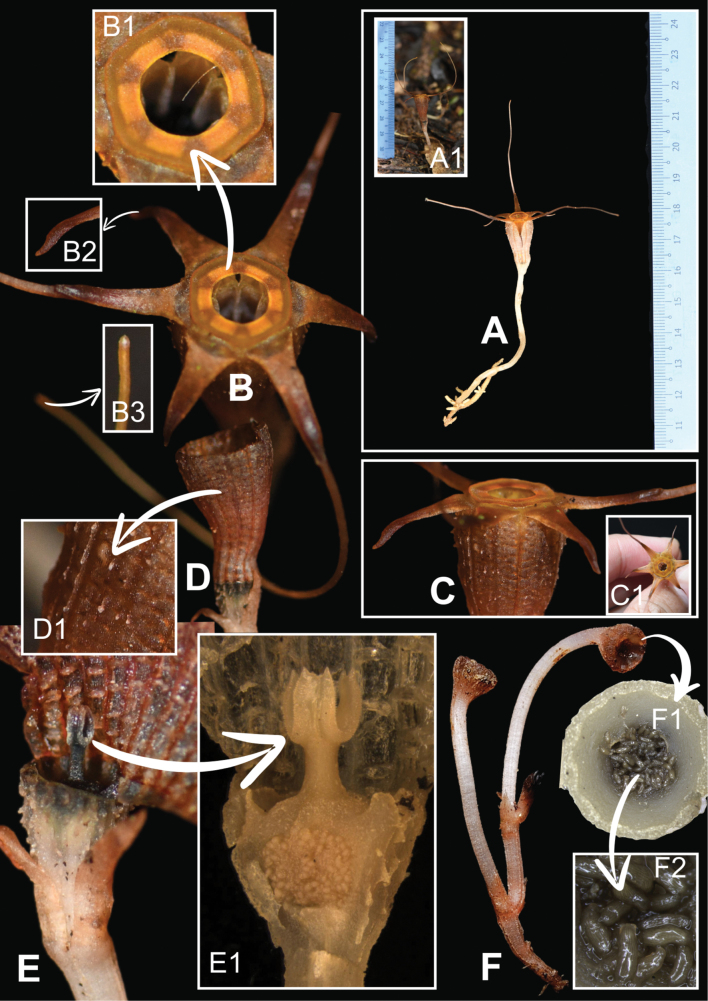
*Thismiaaliasii***A** flowering plant (over-brightened by a camera flash) **B** anthetic flower, top view **B1** annulus, top view **B2** tip of appendage of outer tepal **B3** tip of appendage of inner tepal **C** distal portion of flower, lateral view **C1** flower, top view **D** floral tube (with its apex removed), side view **D1** outer surface of floral tube (showing tiny glands) **E** inner surface of flower tube, pistil and ovary **E1** longitudinal section of ovary and pistil with stigma **F** fruiting plant **F1** seeds in capsule **F2** seeds. Photos by Siti-Munirah (**A, C1**: FRI 79119; **A1, B–E**: FRI 79167; **E1, F1**, **F2**: FRI 91119, spirit material) and Mohamad Alias (**F**: FRI 91119).

#### Description.

Achlorophyllous herb, up to ca. 11 cm tall, mostly glabrous (where not stated otherwise). ***Roots*** vermiform, unbranched, ca. 1.5 mm in diameter, light brown. ***Stem*** erect, up to 56 mm long, 1.8–2 mm in diameter, white-cream to light brown, bearing 1–2 flowers. ***Leaves*** up to 5, spirally arranged (arranged denser at stem base and looser at apex), triangular to narrowly triangular (shorter at stem base and longer and narrower at stem apex), scale-like, apex pointed, margin almost entire to slightly irregularly serrate, up to 7 mm long, ca. 2 mm wide at base, colored similar to stem. ***Flowers*** terminal and solitary, or in 2-flowered terminal inflorescence, actinomorphic, ca. 58 mm long (including ovary, floral tube, tepals and tepal appendages when erected). ***Involucral bracts*** 3, similar to upper leaves, triangular to narrowly triangular, scale-like, acute, with entire margin, 8 mm long, ca. 2.5 mm wide at base, white-brownish/light brown. ***Pedicel*** to 1.5 mm long at anthesis, to ca. 4 mm long after anthesis, white-brownish. ***Floral tube*** obovoid funnel-shaped, 20 mm long, ca. 4 mm wide at base, ca. 8 mm wide at middle, ca. 10 mm wide distally; ***outer surface*** with minute glands, orange to sepia-brown, with 12 darker longitudinal ribs; ***inner surface*** smooth or rough, almost similar color to the outer surface, with transverse bars. ***Tepals*** 6, free, spreading, triangular, apex acute, 7 mm long, ca. 1–1.5 mm wide (ca. 1.5 mm at base), smooth, color similar to flower tube (dark brown at apex, yellow-orange at back), each apically bearing a tentacle-like appendage 0.5 mm wide and narrowing towards the apex; ***appendage of outer tepal*** up to 3.5 mm long, dark brown; ***appendage of inner tepal*** ca. 26–32 mm long, brownish. ***Annulus*** moderately raised, hexagonal in outline, ca. 6 mm in diameter, with ring width ca 1.5 mm, brown-orange, aperture ca. 3 mm in diameter. ***Stamens*** 6, pendulous from annulus; ***filaments*** laterally pale orange, otherwise translucent white; ***connectives*** broad and flattened, fused laterally into a tube, shortly papillose, translucent white, with hairs around thecae, with margins abaxially raised to form conspicuous ribs along the sutures between the connectives, ribs distally slightly protruding beyond the apex of supraconnective; ***interstaminal glands*** conspicuous, placed between bases of lateral appendages; ***supraconnective*** with 3 filiform apical appendages (central appendage ca. 2 mm long and paired appendages ca. 2.4 mm long) and with 2 acute hook-like subapical appendages ca. 0.6 mm long positioned adaxially between the apical appendages and the lateral appendage; ***lateral appendage*** skirt-like, projecting towards the floral tube and not reaching the supraconnective apex, translucent white and brown on sides, lateral margin wavy. ***Ovary*** red, pale cream proximally and blackish distally; unilocular; ***placentas*** 3, free, column-like, arising at ovary base; ovules numerous. ***Style*** dark blackish-orange, ca. 1.2 mm long; stigma ca. 1.4 mm long, papillose, 3-lobed, erect, lobes ± rectangular, bifurcate at the apex, black-greenish. ***Fruit*** dehiscent, cup-shaped, ca. 6 mm high, ca. 7 mm in diameter, pale brownish, darker in the upper part. ***Seeds*** long fusiform ca. 0.75 mm long, ca. 0.25 mm wide.

**Figure 6.**
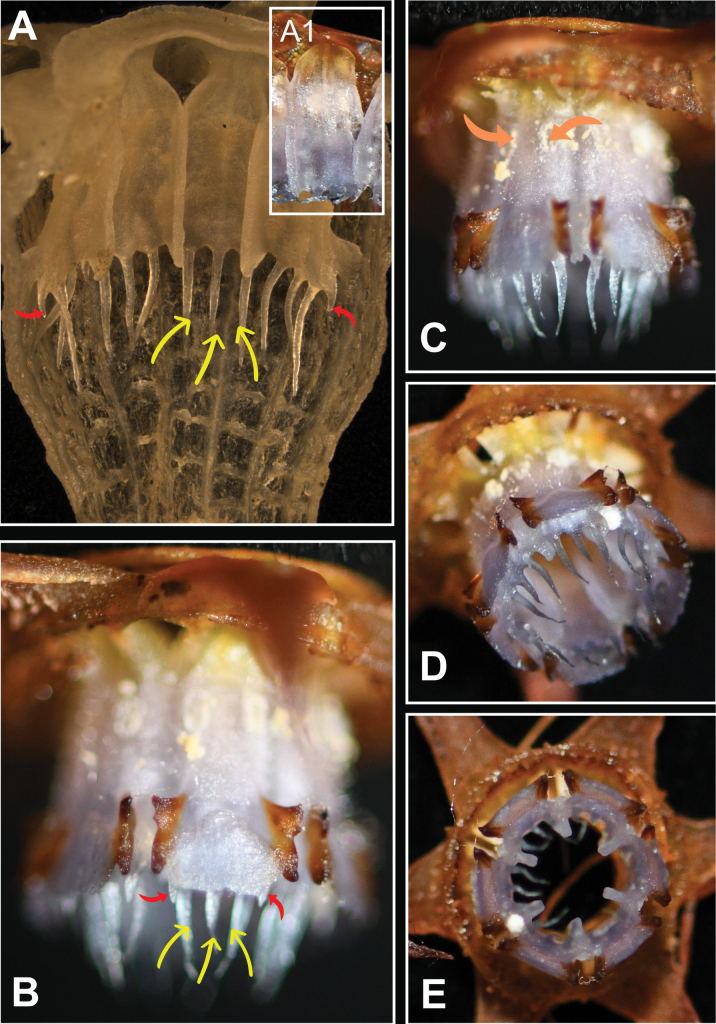
*Thismiaaliasii***A** longitudinally dissected stamen tube showing the margins of individual connectives abaxially raised into conspicuous ribs (spirit material) **A1** stamens, view from inside **B, C** stamens, view from outside **D, E** stamen tube, oblique view from below and view from below (showing apical appendages and longitudinal ribs). Yellow arrows: filiform apical appendages; red arrows: acute hook-like subapical appendage; orange arrows: anthers. All photos by Siti-Munirah (**A**: FRI 91119; **A1, B–E**: FRI 79167).

#### Additional specimens examined.

Malaysia. • Peninsular Malaysia: Terengganu, Hulu Terengganu District, Pasir Raja FR, Chemerong Forest Eco Park, Gunung Chemerong, elev. ca 642 m, 3 October 2019, Mohamad Alias, FRI 91119 (KEP spirit collection, barcode no. SC13202); elev. ca 640 m, 26 July 2023, Siti-Munirah, FRI 79167 (KEP spirit collection, barcode no. SC13203).

#### Distribution.

**Figure 7.**
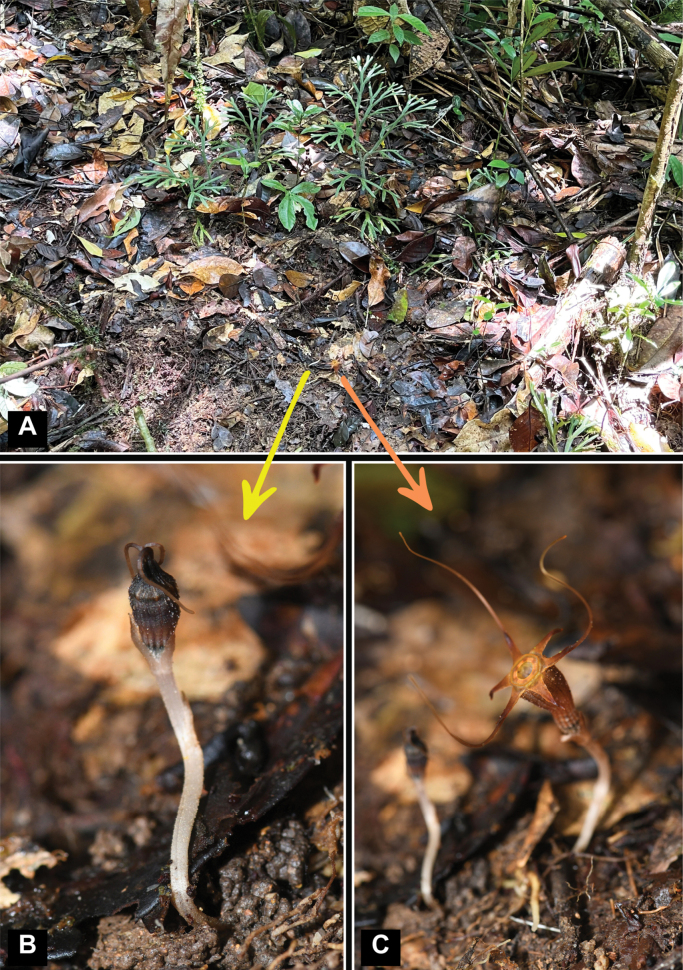
Habitat and habit of *Thismiaaliasii***A** plants in situ **B** plant with flower bud **C** plant with anthetic flower. All photos by Siti-Munirah.

Endemic to Terengganu, Peninsular Malaysia. Currently only known from the type locality, Gunung Chemerong (Figs 2, 3, 7).

#### Ecology.

The species inhabits moist shady areas of upper hill dipterocarp forest on moist soil at elevation of 640 m a.s.l. Flowering and fruiting recorded in July and October. The population was found in moist, shaded areas next to the main hiking trail to the summit of Gunung Chemerong Berembun Langsir at altitudes of 640 m above sea level. The species was found a few hundred meters away from the main river.

#### Etymology.

The species is named after Mr. Alias (the second author), a current ranger of the Terengganu Forestry Department (JPNT), who is also a freelance photographer (known as John Sp) and who was the first to discover the species.

#### Conservation status.

Since 2019, several surveys have been conducted at an area of 4 hectares, but the species was observed only twice, with a total of 5 individuals recorded. The main threat to the population is the degradation of habitat quality due to hiking activities, as the population is found near the main hiking trail which is heavily used by hikers ascending to the summits of Gunung Chemerong Berembun Langsir. Therefore, according to IUCN Red List (IUCN 2024) this species is assessed as Critically Endangered, CR B2ab(iii), D. More surveys are needed to determine the population size of the species.

#### Notes.

*Thismiaaliasii* is easily recognized within the genus by the combination of the following characters: vermiform roots, almost uniform flower coloration (light to dark orange to sepia-brownish red), inner tepals free from each other, unequal tepal appendages (appendages of the outer tepals being shorter than those of the inner tepals), stamens each with 3 long filiform apical appendages, 2 acute subapical appendages and a lateral appendage, and connectives laterally thickened into conspicuous abaxial interstaminal ribs. Within the infrageneric classification of Kumar et al. (2017), *T.aliasii* is assigned here to ThismiasubgenusThismia section ThismiasubsectionOdoardoa Schltr., as long as it has free tepals equal in shape and size. The new species probably belongs to clade 5 defined by Shepeleva et al. (2020), which is characterized by free inner tepals and the presence of appendages of the outer and inner tepals. In addition, the fact that the appendages of the inner tepals are much longer than those of the outer tepals make *T.aliasii* similar to *T.neptunis* Becc., (Sochor at al. 2018), which belongs to T.subgen.Thismia sect. Thismiasubsect.Brunonithismia Jonker according to Kumar et al. (2017), but was recovered as a member of clade 5 by Shepeleva et al. (2020).

The connectives of most *Thismia* species are usually flat and without any processes abaxially, making *T.aliasii* unique not only among T.subsect.Odoardoa but within the entire genus. The only other exceptions are species from T.sect.Geomitra (Becc.) Kumar & S.W. Gale which have ribs in the center of each stamen but differ by coralliform roots and flower mitre.

We summarize Thismiasubsect.Odoardoa to comprise 23 species based on the above-mentioned characteristics and following the classifications of Kumar et al. (2017) and Shepeleva et al. (2020) with additions from Chantanaorrapint et al. (2016), Hroneš et al. (2018), Nishioka et al. (2018), Dančák et al. (2020), Siti-Munirah and Dome (2019), Siti-Munirah et al. (2024), Ya et al. (2024):

##### ﻿A checklist of Thismiasubg.Thismia sect. Thismiasubsect.Odoardoa Schltr., 1921.

Type species: —*Thismiaaseroe* Becc., 1878.

###### ﻿Species included:

1) *T.alba* Holttum ex Jonker, 1948.

2) *T.aliasii* Siti-Munirah, 2025. (this study)

3) *T.annamensis* K.Larsen & Aver., 2007.

4) *T.aseroe* Becc., 1878.

5) *T.bifida* M. Hotta, 1967.

6) *T.bryndonii* Tsukaya, Suetsugu & Suleiman, 2017.

7) *T.chrysops* Ridl., 1895.

8) *T.claviformis* Chantanaorr. & J.Wai, 2016.

9) *T.cornuta* Hroneš, Sochor & Dančák, 2018.

10) *T.domei* Siti-Munirah, 2019.

11) *T.filiformis* Chantanaorr., 2012.

12) *T.fumida* Ridl., 1890.

13) *T.grandiflora* Ridl., 1895.

14) *T.hexagona* Dančák, Hroneš, Kobrlová & Sochor, 2013.

T.hexagonavar.grandiflora Tsukaya, Suleiman & H.Okada, 2014.

15) *T.inconspicua* Sochor & Dančák, 2017.

16) *T.kinabaluensis* T.Nishioka & Suetsugu, 2018.

17) *T.lauriana* Jarvie, 1996.

18) *T.malayana* Siti-Munirah, Hardy-Adrian, Mohamad-Shafiq & Irwan-Syah, 2024.

19) *T.mullerensis* Tsukaya & H.Okada, 2005.

20) *T.ophiuris* Becc., 1878.

21) *T.ornata* Dančák, Hroneš & Sochor, 2020.

22) *T.pallida* Hroneš, Dančák & Rejzek, 2018.

23) *T.racemosa* Ridl., 1915.

Ten species of the subsection occur in Peninsular Malaysia (*T.alba*, *T.aseroe*, *T.chrysops*, *T.domei*, *T.fumida*, *T.grandiflora*, *T.malayanaT.ornata*, *T.racemosa* and *T.aliasii*), with five of them (*T.alba*, *T.aliasii*, *T.aseroe*, *T.domei* and *T.ornata*) occurring in the state of Terengganu. Whereas most of these species are characterized by tepal appendages equal in size and shape, in *T.aliasii* the appendages of the inner tepals are longer than those of the outer tepals. In addition, the stamen connectives laterally raised to form a conspicuous interstaminal rib are a unique feature of *T.aliasii* that is not found in the other species of T.subsect.Odoardoa.

## Supplementary Material

XML Treatment for Thismia aliasii

## ﻿Additional information

### Author ORCIDs

Mat Yunoh Siti-Munirah https://orcid.org/0000-0002-5062-9988

### Data availability

All of the data that support the findings of this study are available in the main text.
